# The risk prediction models for sarcopenia in older adults: a systematic review and critical appraisal

**DOI:** 10.3389/fpubh.2026.1751954

**Published:** 2026-01-29

**Authors:** Taiping Lin, Hualong Liao, Lin Su, Ping Xu, Xiangping Tu, Lunzhi Dai, Jufeng Luo, Qiao Xiang, Ning Ge, Jirong Yue

**Affiliations:** 1 Department of Geriatrics and National Clinical Research Center for Geriatrics, West China Hospital of Sichuan University, Chengdu, Sichuan, China; 2 Department of Geriatrics and National Clinical Research Center for Geriatrics, Frontiers Science Center for Disease-related Molecular Network, West China Hospital, Sichuan University, Chengdu, Sichuan, China; 3 Department of Biomedical Engineering, Sichuan University Library, Chengdu, Sichuan, China; 4 Department of Laboratory Medicine, Clinical Laboratory Medicine Research Center, West China Hospital, Sichuan University, Chengdu, Sichuan, China; 5 National Clinical Research Center for Geriatrics and Department of Laboratory Medicine, State Key Laboratory of Biotherapy, West China Hospital, Sichuan University, Chengdu, China; 6 Department of Geriatrics, West China Xiamen Hospital of Sichuan University, Xiamen, China

**Keywords:** critical appraisal, older adults, prediction model, sarcopenia, systematic review

## Abstract

**Introduction:**

Reliable sarcopenia risk prediction models are essential for identifying older adults who are currently non-sarcopenic but at risk of developing sarcopenia in the future, thereby enabling early and personalized prevention strategies. However, the prediction models for sarcopenia have not yet been systematically evaluated. This systematic review aimed to conduct a comprehensive overview and critical appraisal of current sarcopenia risk prediction models.

**Methods:**

We conducted a systematic search across MEDLINE, Embase, Cochrane Library, and SCI-EXPANDED. Eligible primary studies on sarcopenia prediction models were identified based on the CHARMS checklist (CHecklist for critical Appraisal and data extraction for systematic Reviews of prediction Modelling Studies). The Prediction model Risk Of Bias Assessment Tool (PROBAST) was applied to evaluate risk of bias and clinical applicability.

**Results:**

Twenty-six sarcopenia prediction models were identified, mostly targeting community-dwelling older adults or patients. Twenty-three studies developed diagnostic prediction models, while only three studies established sarcopenia prognostic models. Age, BMI, calf circumference and gender were most frequently utilized predictors. Despite reported discriminative performance ranging from moderate to excellent (AUC > 0.70), 96.1% of prediction models exhibited high risk of bias due to significant methodological shortcomings, suggesting that model performance might be overestimated. Moreover, most existing prediction models were diagnostic study design, limiting their ability to predict the future risk of sarcopenia development.

**Conclusion:**

Most existing sarcopenia prediction models demonstrated moderate to high discriminatory performance. However, due to their predominantly diagnostic study design and high risk of bias, these models cannot yet be broadly recommended for routine clinical application in the early identification of high-risk older adults with sarcopenia. Future studies are needed to develop and externally validate practical, accurate prognostic sarcopenia models to fulfill sarcopenia early prevention.

**Systematic review registration:**

The protocol has been registered on the Open Science Framework (10.17605/OSF. IO/BFDK6).

## Introduction

The rise in global life expectancy has led to a growing aging population, highlighting age-related conditions like sarcopenia as pressing public health challenges (1). Sarcopenia, defined as the progressive loss of skeletal muscle mass, strength, and physical performance, is closely associated with adverse health outcomes, including frailty, falls, disability, hospitalizations, and mortality (2, 3). While muscle strength and physical performance are effective for identifying existing sarcopenia, they primarily capture current functional status rather than forecast future risk. Predictive models integrate multiple demographic, clinical, and physiological factors to estimate the likelihood of developing sarcopenia before overt symptoms arise. Such prognostic tools can facilitate early, individualized interventions and enable precision prevention strategies in geriatric care (4–6).

In recent years, risk prediction models for sarcopenia have emerged as promising tools to support early detection and informed clinical decision-making (7–10). These models estimated an individual’s likelihood of developing sarcopenia by integrating demographic, clinical, and functional indicators. Despite growing progress has been made in this research filed, current sarcopenia prediction models varied considerably in study design, population demographics, predictor variables, and outcome definitions. Moreover, many models were tailored for diagnostic purposes rather than to estimate future sarcopenia risk (8–10). This limited their utility for stratified risk evaluation and personalized management—approaches essential for effective preventive strategies in aging populations. The lack of external validation, improper handling of missing data and continuous predictors, and insufficient reporting of model equations and performance metrics may contribute to high risk of bias according to the PROBAST tool (Prediction model Risk Of Bias ASsessment Tool (11). Additionally, several models depend on predictors that may not be feasible or cost-effective for routine implementation, particularly in primary care or low-resource settings.

Given these challenges, there is a pressing need to systematically evaluate the current landscape of sarcopenia prediction models. However, the overall assessment of sarcopenia prediction model has not yet examined. Of note, such an evaluation can help identify methodological gaps, highlight opportunities for model refinement, and guide the development of robust, practical, and precise tools for identifying high-risk older adults for developing sarcopenia and facilitating individualized management for sarcopenia (12, 13). The aim of this study is to conduct a systematic review and critical appraisal of existing sarcopenia prediction models using the PROBAST tool.

## Methods

This systematic review adhered to the guidelines outlined in the CHARMS framework (CHecklist for critical Appraisal and data extraction for systematic Reviews of prediction Modelling Studies) (14–16) and followed the principles outlined in the PRISMA 2020 statement (Preferred Reporting Items for Systematic Reviews and Meta-Analyses) (17). The protocol has been registered on the Open Science Framework (10.17605/OSF. IO/BFDK6).

### Literature search strategy

The search strategy was developed and subsequently refined in collaboration with a medical librarian specialist (P. X.) and a senior expert in systematic reviews (J. Y.). Using the Ovid SP platform, we searched four major biomedical databases: MEDLINE, Embase, the Cochrane Central Register of Controlled Trials, and the Science Citation Index Expanded. The database-specific search strategies are provided in Supplementary Table S1. An updated search was conducted up to 29 September 2025. Additionally, reference lists of included studies were manually screened to ensure comprehensive coverage of relevant literature.

### Inclusion and exclusion criteria

This review covered all relevant studies that have developed multivariable prediction models, tools, indices, or scoring systems (defined as models containing at least two predictors) aimed at assessing the risk of sarcopenia in older adults. We specifically searched for studies related to the development of sarcopenia prediction models. Studies that solely evaluated the diagnostic accuracy or external validation of existing screening tools or scores, without model development, were not included in the primary synthesis. We structured the inclusion criteria for this review based on the PICOTS framework (Population, Intervention, Comparator, Outcomes, Timing, and Setting), commonly used in systematic reviews of prediction models (Supplementary Table S2): (1) Population: Studies involving older adults from diverse settings, including community-dwelling individuals, nursing homes, hospitals, and those with chronic diseases or cancer; (2) Intervention: Models predicting the presence of sarcopenia (diagnostic models) or the future risk of sarcopenia development (prognostic models); (3) Comparator: No specific model comparison was required; (4) Outcomes: The outcome of interest was the presence of sarcopenia at a given time point or the risk of developing sarcopenia over a future time period; (5) Timing: No specific time restrictions were imposed on the measurement of predictors or outcomes; (6) Setting: Community, primary care, long-term care facilities, or hospital settings.

To reduce heterogeneity across studies, diagnostic criteria for sarcopenia were restricted to widely accepted definitions, including AWGS (2014/2019) (2, 18) and EWGSOP (2010/2018) (7, 19), which defined sarcopenia as low muscle mass (LMM) combined with low muscle strength (LMS) and/or low physical performance (LPP). Studies were excluded if they: (1) Lacked clear diagnostic criteria for sarcopenia or defined it using a single parameter; (2) Systematic reviews, meta-analyses, commentaries, or methodological studies; (3) Focused on genetic/biological markers of sarcopenia or sarcopenia screening scales; (4) Predictive equation or the full text was unavailable; (5) Non-English publications; (6) Duplicated publications.

### Study selection

Two reviewers (T. L. and N. S.) independently screened the titles and abstracts to identify potentially relevant studies. The study selection process followed three main phases: (1) Title and Abstract Screening: All retrieved references were imported into EndNote software, where duplicates were automatically removed. Two independent reviewers screened the titles and abstracts of the remaining studies against predefined inclusion criteria to exclude irrelevant articles. A third reviewer cross-checked the results to ensure consistency. Discrepancies were resolved through facilitated discussions led by the third reviewer. When consensus could not be reached, the issue was escalated to the full research team for resolution. (2) Full-Text Screening: Studies passing preliminary screening underwent detailed full-text review according to the inclusion and exclusion criteria. Two reviewers independently assessed the main texts and Supplementary materials to confirm eligibility. The extracted data were cross-verified for accuracy. Inter-rater agreement during full-text screening was quantified using the Kappa statistic, which evaluated observer agreement while accounting for chance. The Kappa statistic, categorized as poor (<0), mild (0.20–0.40), moderate (0.41–0.60), high (0.61–0.80), and substantial (0.81–1.00), confirmed strong agreement among reviewers. (3) Handling Incomplete Data: For studies with missing or incomplete data, corresponding authors were contacted via email or phone. Any disagreements during the process were resolved through discussion with a third researcher (J. Y.).

### Data extraction and assessment of predictive performance

Data extraction adhered to a standardized form developed using the CHARMS checklist (14). Two reviewers (H. L. and P. X.) independently extracted data, and a third reviewer cross-verified the extracted information. The key elements captured were: (1) General information: First author, year of publication, journal, study type, and geographic location. (2) Study details: Data source, setting, population, and sample size. (3) Model specifics: Predictors, sarcopenia definition, model development/validation approach, performance metrics, and presentation format. (4) Methodological details: Handling of missing data and use of continuous variables. To guard against overly optimistic evaluations of prediction model performance, data from internal or external validation datasets were prioritized. If validation data were unavailable, performance metrics were conservatively extracted from the lowest-performing subgroup in the training dataset.

To evaluate the predictive ability of each model, we extracted the key indicators related to the performance of discrimination, calibration and classification. To evaluate how well each model distinguishes between individuals who develop sarcopenia and those who do not, we used the area under the receiver operating characteristic curve (AUC) as the primary measure of discrimination (15). In the absence of validation data, we extracted the minimum reported AUC value as the most conservative estimate. For research that proposed multiple models, priority should be given to the model with the most complete information, including the complete prediction equation. The AUC values were categorized as poor (0.50–0.59), average (0.60–0.69), good (0.70–0.79), very good (0.80–0.89), and excellent (≥0.90) (15). Calibration assessed the consistency between the predicted and observed results, using calibration intercepts and slopes for evaluation (20). Extract the classification performance when available to provide a comprehensive understanding of the diagnostic accuracy of the model, such as sensitivity, specificity, accuracy, positive predictive value, and negative predictive value.

### Risk of Bias assessment and critical appraisal

We systematically applied the Prediction model Risk Of Bias ASsessment Tool (PROBAST) to evaluate both the risk of bias and the applicability of each prediction model (Q. X. and J. L.). PROBAST assessed four key domains—participants, predictors, outcomes, and analysis—using 20 signaling questions to evaluate methodology related to study design, conduct, and data analysis (11). Each question was categorized as “Yes,” “Probably Yes,” “Probably No,” “No,” or “No Information.” The overall risk of bias for each domain was classified as low, high, or unclear. A domain was judged to be at high risk of bias if one or more signaling questions are answered as “No” or “Probably No.” A low-risk domain required all questions to be “Yes” or “Probably Yes.” A prediction model was rated as having low overall risk of bias only if all four domains are assessed as low risk. If any domain was rated as high risk, the overall judgment is high risk. If at least one domain had an unclear risk of bias, and all other domains were rated as low risk, the model was categorized as having an unclear overall risk of bias. In addition to the bias assessment, the applicability of the prediction model was evaluated based on the first three domains—participants, predictors, and outcomes. Applicability concerns were classified as low, high, or unclear based on expert judgment, without the use of specific signaling questions. Any discrepancies in judgments were resolved through discussion among the reviewers to ensure consensus. By utilizing PROBAST, it was possible to critically appraise the methodological rigor and applicability of prediction models, offering a scientifically robust foundation for interpreting the reliability and generalizability of the research findings.

### Statistical analysis

Considering the significant heterogeneity of the included studies in terms of the study population, study design, model development and validation methods, and the final included predictor subsets, we did not conduct quantitative synthesis of the reported predictive performance (AUC values), such as summary effect size estimation, subgroup analysis, sensitivity analysis, or publication bias assessment (15, 21). Instead, we used Stata/SE version 15.1 (StataCorp LP, College Station, TX, USA) to summarize the reported AUC values of the prediction model in the original study and generate forest plots to represent the model performance comprehensively (15). Furthermore, we used Microsoft Excel to construct a bar chart based on the PROBAST assessment to illustrate the overall risk of bias and applicability ratings of the included prediction models. The bar chart was also used to show the most common predictors in the model. These findings provided practical references for the selection of variables when developing and validating high-quality sarcopenia prediction models in the future. Furthermore, we summarized the baseline characteristics of all the included models. Continuous variables were reported as the median of the quartile range (IQRs), while categorical variables were expressed as frequency and percentage.

## Results

### Study selection

**Figure 1:**
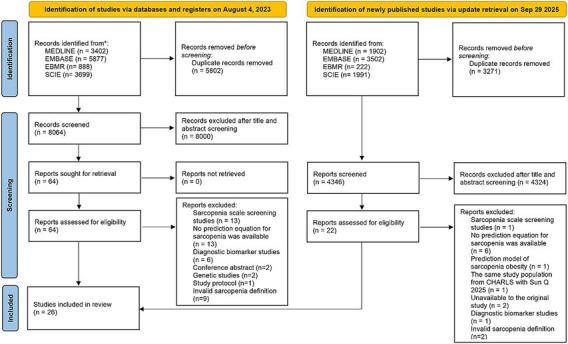
Flowchart of search results and study selection.

A comprehensive literature search on August 4, 2023 identified 13,866 records. After removing 5,802 duplicates, 8,064 records were screened, resulting in 64 full-text reviews, of which 18 studies met the inclusion criteria (Figure 1) (9, 22–38). Additionally, the literature search was updated on 29 September 2025 and another 8 studies were included (Figure 1) (39–46). Exclusion reasons for full-text reviews were detailed in Supplementary Table S3. Inter-reviewer agreement was quantified using the Kappa statistic, resulting in a value of 0.80, indicating strong agreement between reviewers.

### Basic characteristics of included studies

The included studies, summarized in Table 1, involved 33,273 participants across China (*n* = 21), Japan (*n* = 3), South Korea (*n* = 1), and Iran (*n* = 1). Thirteen models (50%) were developed in community-based populations, while the remaining studies were hospital-based patients with chronic disease. Similarly, half targeted general older adults, while others focused on specific populations, including dialysis patients (*n* = 4), diabetes mellitus (*n* = 5), stroke (*n* = 1), rheumatoid arthritis (*n* = 1) and cancer patients (*n* = 1). Most models (*n* = 23) were derived from cross-sectional studies for diagnostic purposes, and only three cohort-based models focused on prognosis. Sample sizes ranged from 137 to 4,610, with sarcopenia prevalence between 5.8 and 22.5%. Except for three studies, all sampled participants were aged 60 years or older.

**Table 1:** Characteristics of the included studies.

Author/year	Population	Model design	Missing data handling	Continuous data handling	Modelling method	Internal validation technique	External validation	Candidate predictors	Model discrimination	Model calibration	Model presentation	Sarcopenia Criteria
Mo et al. 2022 (9)	1,050 community-dwelling adults aged 60 years or older in China	Diagnostic model	Not reported	Categorical/dichotomous	Multivariate logistical regression model	Cross validation	None	Age, BMI, marital status, regular physical activity habit, dietary diversity score, uninterrupted sedentary time	AUC_DS_: 0.827 (95% CI 0.792–0.860); AUC_VS_: 0.755 (95% CI 0.680–0.837)	Hosmer-Lemeshow tests and Calibration plot	Nomogram risk chart	AWGS 2019 (LMM; LMS; LGS)
Cai et al. 2022 (22)	615 hemodialysis patients with an average age of 60.7 years in China	Diagnostic model	Replaced by the mean of the complete continuous data	Categorical/dichotomous	Multivariate logistical regression model	Bootstrapping	None	Age, C-reactive protein, MAC, BMI, serum phosphorus	AUC_DS_: 0.869 (95% CI 0.822–0.915); AUC_VS_: 0.832 (95% CI 0.765–0.900)	Calibration plot	Nomogram risk chart	AWGS 2019 (LMM; LMS; LGS)
Kera et al. 2022 (27)	627 community-dwelling adults aged 65–85 years in Japan	Diagnostic model	Not reported	Continuous	Multivariate logistical regression model	Not reported	None	Age, ground reaction force during sit-to-stand motion	AUC_Men_: 0.906 (95% CI 0.858–0.954); AUC_Women_: 0.858 (95% CI 0.808–0.908)	Not reported	Prediction formula	AWGS 2019 (LMM; LMS; LGS)
Ishii et al. 2014 (25)	1971 community-dwelling adults aged 65 years or older in Japan	Diagnostic model	No missing values	Continuous	Multivariate logistical regression model	Bootstrapping	None	Age, grip strength, and CC	AUC_Men_: 0.939 (95% CI 0.918–0.958); AUC_Women_: 0.909 (95% CI 0.887–0.931)	Hosmer-Lemeshow tests	Risk chart	EWGSOP (LMM; LMS; LGS)
Shafiee et al. 2021 (28)	2,211 community-dwelling adults aged 60 years or older in Iran	Diagnostic model	Not reported	Continuous	Multivariate logistical regression model	Cross validation	None	Age, weight, and CC	AUC_Men_: 0.82 (95% CI 0.79–0.86), and AUC_Women_: 0.87 (95% CI 0.84–0.90) in the DS; AUC_Men_: 0.84 (95% CI 0.80–0.89), and AUC_Women_: 0.91 (95% CI 0.88–0.94) in the VS	Not reported	Prediction formula	EWGSOP 2 (LMM; LMS; LGS)
Chen et al. 2020 (18)	137 older patients with an average age of 65.7 years who underwent patellar fracture in China	Diagnostic model	Not reported	Continuous	Multivariate logistical regression model; LASSO; SVM	Bootstrapping	None	Age, BMI, diabetes, postoperative rehabilitation, frequency of outdoor exercise, surgical method used, presence or absence of open fracture, removal of internal fixation	AUC: 0.88 (95%CI 0.81–0.95)	Calibration plot	Nomogram	AWGS 2014 (LMM; LGS)
Shin et al. 2023 (29)	1,021 community-dwelling adults aged 70 years or older in the Republic of Korea	Prognostic model	Exclusion of patients with missing data	Continuous	Ridge regression analysis	Not reported	None	GDF15, cystatin C, DHEA, hemoglobin, myostatin, AST/ALT ratio, eGFR, and BUN	AUC: 0.71 (95%CI 0.67–0.75)	Not reported	Not reported	AWGS 2019 (LMM; LGS or SPPB score of ≤9 or 5-CST time of ≥12 s)
Du et al. 2022 (32)	805 patients with an average age of 53.8 years undergoing maintenance hemodialysis in China	Diagnostic model	Not reported	Continuous	Multivariate logistical regression model	Not reported	None	Age, BMI, CC, serum creatinine	AUC_DS_: 0.922 (95% CI 0.899–0.946); AUC_VS_: 0.913 (95% CI 0.870–0.956)	Hosmer-Lemeshow tests	Nomogram	AWGS 2019 (LMM; LMS; LGS)
Kamitani et al. 2021 (26)	1,334 orthopaedic patients with an average age of 69.5 years in Japan	Diagnostic model	Exclusion of patients with missing data	Categorical/dichotomous	Multivariate logistical regression model	Bootstrapping	None	Age, BMI, strength, and thin	AUC: 0.77 (95% CI 0.71–0.83)	Not reported	Risk chart	AWGS 2019 (LMM; LMS; LGS)
Zhang et al. 2023 (31)	4,610 community-dwelling adults aged 50 year or older in China	Diagnostic model	Exclusion of patients with missing data	Continuous	Machine learning	Cross validation	Yes	Age, weight, TST, CC, MAC, AST/ALT, itchy skin, syncope, MCANEE, MCAILL, LFPHY, LFIACT	AUCex-_VS_: 0.722 (95% CI 0.65–0.81)	Not reported	Not reported	AWGS 2019 (LMM; LMS; LGS or SPPB)
Yu et al. 2023 (37)	1,131 hospitalized patients (mean age 62.67 ± 11.25 years) with type 2 diabetes mellitus in China	Diagnostic model	Multiple imputation techniques	Continuous	LASSO; Multivariate logistical regression model	Bootstrapping	Yes	Age, gender, BMI, waist-hip ratio, and heart rate	AUC_DS_: 0.908 (95% CI 0.886–0.928); AUC_VS_: 0.904 (95% CI 0.868–0.941)	Calibration plot	Nomogram	AWGS 2014 (LMM; LMS; LGS)
Zhang and Zhu 2023 (38)	359 patients with colorectal cancer aged 60 year or older in China	Diagnostic model	Not reported	Not included	Multivariate logistical regression model	Random split	None	Smoking history, drinking history, diabetes, TNM stage, nutritional status, and physical activity	AUC_DS_: 0.971 (95% CI 0.954–0.988); AUC_VS_: 0.922 (95% CI 0.820–1.000)	Calibration plot; and Hosmer-Lemeshow tests	Nomogram	AWGS 2019 (LMM; LMS; LGS)
Xie et al. 2023 (35)	757 hemodialysis patients with an average age of 60.4 years in China	Diagnostic model	Not reported	Continuous	Multivariate logistical regression model	Bootstrapping	None	Age, gender, weight, and grip strength	AUC_DS_: 0.929 (95% CI 0.904–0.953); AUC_VS_: 0.955 (95% CI 0.931–0.979)	Calibration plot	Nomogram	AWGS 2019 (LMM; LMS; LGS)
Yin et al. 2023 (36)	180 community-dwelling adults aged 60 years or older in China	Diagnostic model	Multiple imputation techniques	Continuous	LASSO; Multivariate logistical regression model	Bootstrapping	None	Age, albumin, blood urea nitrogen, grip strength, and CC	AUC_DS_: 0.90 (95% CI 0.85–0.95); AUC_VS_: 0.92 (95% CI 0.83–1.00)	Calibration plot; and Hosmer-Lemeshow tests	Nomogram	AWGS 2019 (LMM; LMS; LGS)
He et al. 2023 (33)	1,125 patients with type 2 diabetes in China with an average age of 60 years and above	Diagnostic model	Not reported	Categorical/dichotomous	Multivariate logistical regression model	Not reported	Yes	Age, gender, BMI, total energy intake per day, and the proportion of calories supplied by protein	AUC_DS_: 0.806 (95% CI 0.741–0.872); AUC_VS_: 0.836 (95% CI 0.781–0.892)	Hosmer-Lemeshow tests	Risk chart	AWGS 2014 (LMM; LMS)
Wu et al. 2022 (34)	1,125 patients undergoing peritoneal dialysis (mean age 54.2 ± 8.89 years) in China	Diagnostic model	Exclusion of patients with missing data	Not reported	Multivariate logistical regression model; LASSO; Machine learning	Not reported	None	BMI, grip strength, total body water value, irisin, extracellular water/total body water, fat-free mass index, phase angle, albumin/globulin, blood phosphorus, triglyceride, total cholesterol, and prealbumin	AUC_DS_: 0.82 (95% CI 0.67–1.00)	Not reported	Not reported	AWGS 2019 (LMM; LMS; 5-CST)
Huang et al. 2023 (24)	966 community-dwelling older adults with an average age of 71.0 years in China	Diagnostic model	Exclusion of patients with missing data	Categorical/dichotomous	Multivariate logistical regression model	Bootstrapping	None	Age, BMI, CC, congestive heart failure, and chronic obstructive pulmonary disease	AUC_DS_: 0.930 (95% CI 0.907–0.952); AUC_VS_: 0.897 (95% CI 0.858–0.936)	Hosmer-Lemeshow tests	Nomogram	AWGS 2019 (LMM; LMS; LGS)
Wang et al. 2022 (30)	1,417 community-dwelling older adults with an average age of 63.3 years in China	Diagnostic model	Not reported	Categorical/dichotomous	Multivariate association with linear models, and the random forest mode	Random split	None	*Desulfovibrio piger*, *Clostridium symbiosum*, Hungatella effluvii, *Bacteroides fluxus*, Absiella innocuum, and *Clostridium citroniae*	AUC_VS_: 0.852 (95% CI 0.81–0.89)	Not reported	Not reported	AWGS 2019 (LMM; LMS; LGS)
Zhang et al. (46)	300 older adults patients with Type 2 Diabetes Mellitus in tertiary care hospitals in China	Diagnostic model	Exclusion of patients with missing data	Continuous	LASSO; Multivariate logistical regression model	Not reported	Yes	Age, gender, HbA1c, serum albumin, thyroid function, and physical activity level	AUC_DS_: 0.891 (95% CI 0.914–0.963); AUC_VS_: 0.868 (95% CI 0.935–0.972)	Calibration plot	Nomogram	AWGS 2019 (LMM; LMS)
Sun et al. (43)	2,197 individuals who participated in a longitudinal study from 2011 to 2013 in China	Prognostic model	Exclusion of patients with missing data	Continuous	LASSO; Multivariate logistical regression model	Random split	None	Age, BMI, female, memory-related diseases, arthritis or rheumatism, shorter sleep duration, and lower education levels	AUC: 0.849 (95% CI 0.821–0.878)	Hosmer-Lemeshow tests	Prediction formula	AWGS 2019 (LMM; LMS; LGS)
Yan et al. (45)	794 patients with stroke in China	Diagnostic model	Exclusion of patients with missing data	Continuous	LASSO; Multivariate logistical regression model and machine learning	Bootstrapping	Yes	Age, diabetes, limb dysfunction, tube feeding, BMI, NIHSS score and C-reactive protein	AUC_DS_: 0.805; AUC_VS_: 0.816	Calibration plot; and Hosmer-Lemeshow tests	Nomogram; Risk calculator	AWGS 2019 (LMM; LMS; 5-CST time of ≥12 s)
Qiao et al. (41)	2,131 middle-aged and older adults with diabetes mellitus from the CHARLS database collected in 2015	Diagnostic model	Exclusion of patients with missing data	Continuous	LASSO; Multivariate logistical regression model	Random split	None	Age, residence, body mass index, diastolic blood pressure, cognitive function, activities of daily living, peak expiratory flow and hemoglobin	AUC_DS_: 0.867(95% CI 0.847–0.887); AUC_VS_: 0.849 (95% CI 0.816–0.883)	Calibration plot	Nomogram	AWGS 2019 (LMM; LMS; LGS)
Lin et al. 2023 (47)	1,042 community-dwelling older adults aged 60 years or older in China	Prognostic model	Multiple imputation techniques	Categorical/dichotomous	Multivariate logistical regression model	Bootstrapping	Yes	Age, gender, BMI, low physical activity, malnutrition, pain, calf circumference	AUC_DS_: 0.87(95% CI 0.83–0.90); AUC_VS_: 0.85 (95% CI 0.81–0.88)	Calibration plot	Nomogram; Risk calculator	AWGS 2019 (LMM; LMS; LGS)
Wang et al. 2024 (48)	1,434 older patients (≥ 60 years) diagnosed with type 2 diabetes mellitus	Diagnostic model	Exclusion of patients with missing data	Continuous	LASSO; Multivariate logistical regression model	Random split	None	Age, BMI, diabetic duration, glycated hemoglobin A1c, 25 (OH)Vitamin D, nephropathy, neuropathy, nutrition status, osteoporosis	AUC_DS_: 0.800(95% CI 0.773–0.828); AUC_VS_: 0.796 (95% CI 0.755–0.838)	Calibration plot; and Hosmer-Lemeshow tests	Nomogram	AWGS 2019 (LMM; LMS; LGS)
Li et al. (39)	3,454 older adults enrolled in the CHARLS database in 2015	Diagnostic model	Exclusion of patients with missing data	Continuous	LASSO; Multivariate logistical regression model	Not reported	None	Sex, BMI, Mean Systolic Blood Pressure, Mean Diastolic Blood Pressure and pain	AUC_DS_: 0.77(95% CI 0.75–0.79); AUC_VS_: 0.76 (95% CI 0.73–0.79)	Calibration plot; and Hosmer-Lemeshow tests	Nomogram	AWGS 2019 (LMM; LMS; LGS or 5-CST time of ≥12 s)
Qu et al. (42)	480 patients with rheumatoid arthritis	Diagnostic model	Exclusion of patients with missing data	Categorical/dichotomous	LASSO; Multivariate logistical regression model	Random split	None	BMI, disease duration, hemoglobin, grip strength	AUC_DS_: 0.915(95% CI 0.880–0.950); AUC_VS_: 0.907 (95% CI 0.855–0.959)	Calibration plot	Nomogram	AWGS 2019

AUC, area under the receiver operator characteristic curve; DS, the development set; VS, the validation set; Lasso, the least absolute shrinkage model and selection operator regression; SVM, the Support Vector Machine algorithm; GDF 15, growth differentiation factor 15; DHEA, dehydroepiandrosterone; AST, aspartate aminotransferase; ALT, alanine aminotransferase; BUN, blood urine nitrogen; eGFR, estimated using the Chronic Kidney Disease Epidemiology Collaboration (CKD-EPI) creatinine equation; SPPB, The Short Physical Performance Battery; 5-CST, The five-times chair stand test; BMI, body mass index; TST, triceps skinfold thickness; CC, calf circumference; MAC, mid-upper arm circumference; AST/ALT, the ratio of alanine aminotransferase to aspartate aminotransferase; MCANEE, have you had any illness in the last year that required care; MCAILL, did you see a doctor in the last 2 weeks because of illness or discomfort; LFPHY, has your physical activity decreased in the last month; LFIACT, have you engaged in household chores of moderate activity in the past 2 weeks; TNM, tumor-node-metastasis stage. All listed studies above were reporting the development of a new multivariable prediction model for sarcopenia.

### Diagnostic criteria for sarcopenia

Significant variations existed in the diagnostic methods and cutoff values for sarcopenia (Supplementary Table S4). Most studies assessed LMM using bioelectrical impedance analysis (BIA, *n* = 18), while four employed dual-energy X-ray absorptiometry (DXA). Muscle strength was consistently measured via handgrip strength, whereas physical performance was evaluated using diverse methods, including 6-meter (*n* = 14), 4-meter (*n* = 3), and 5-meter (*n* = 2) walk tests. Four studies incorporated multiple measures, such as gait speed, Short Physical Performance Battery (SPPB), or chair stand tests. Diagnostic criteria for sarcopenia definition also varied: 21 used AWGS 2019 standards, 3 used AWGS 2014, 1 used EWGSOP1, and 1 adopted EWGSOP2.

### Evaluation of predictors

**Figure 2:**
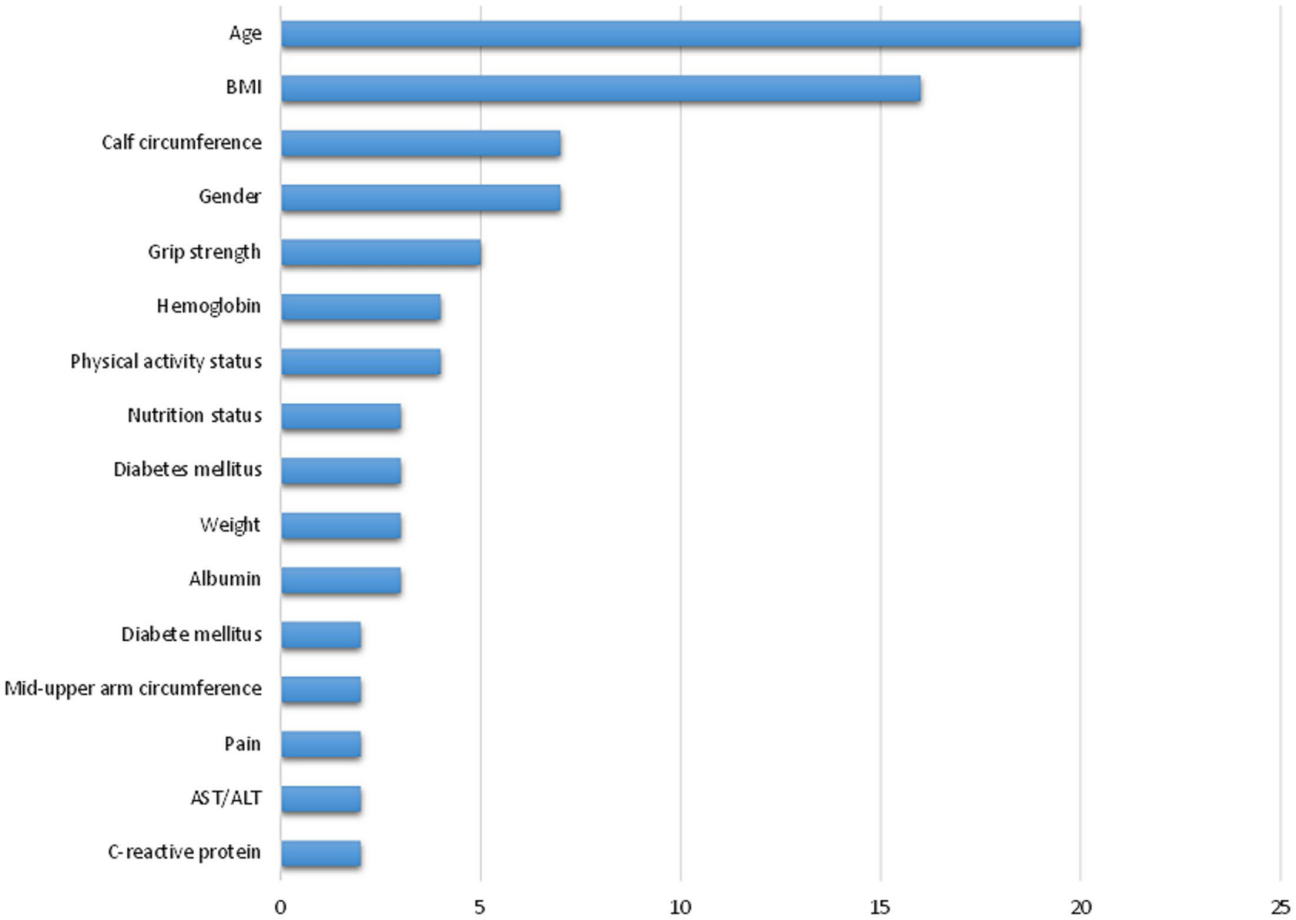
The frequency of most common predictors used by at least two prediction models.

Over 50 predictors were identified and grouped into demographics, health-related risks, chronic diseases, socioeconomic factors, and body composition. Among these, eight predictors were used in multiple models, including age (*n* = 20), BMI (*n* = 16), gender (*n* = 7), calf circumference (*n* = 7) and so on. Age was the most frequently integrated predictor, reflecting its strong association with sarcopenia (Figure 2).

### Handling of missing data and continuous variables

Nine studies did not report handling of missing data. Of the remaining 17, 12 excluded incomplete records, three applied multiple imputation, one used mean substitution, and one explicitly stated no missing data. For continuous variables, 16 models retained continuous predictors, while 8 models classified continuous variables into binary or multi-level categorical variables, potentially reducing predictive power. One model provided unclear information, and another treated all variables as categorical (Table 1).

### Model development and validation

Most models (*n* = 12, 46.1%) were developed using multivariable logistic regression. Other techniques included machine learning (*n* = 1), ridge regression (*n* = 1), and combined methods like LASSO and logistic regression (*n* = 12). Internal validation was conducted in 19 studies via bootstrapping (*n* = 10), cross-validation (*n* = 3), and data splitting (*n* = 6). We did not identify any separate external validation reports for already published sarcopenia models. However, 7 studies lacked internal validation. External validation was performed in only 6 studies (Table 1).

### Model performance and presentation

All 26 models reported discrimination via AUC values, 20 models achieving AUC > 0.80 (strong discrimination) and the remaining 6 within 0.70–0.80 (acceptable). Calibration metrics were inconsistent—19 studies reported calibration methods (e.g., Hosmer-Lemeshow test, calibration curves). Models were presented as regression formulas (*n* = 3), nomograms (*n* = 12), risk score tables (*n* = 3), or combined formats (*n* = 4); 4 studies did not report presentation formats (Table 1).

### Risk of Bias and applicability assessment

**Figure 3:**
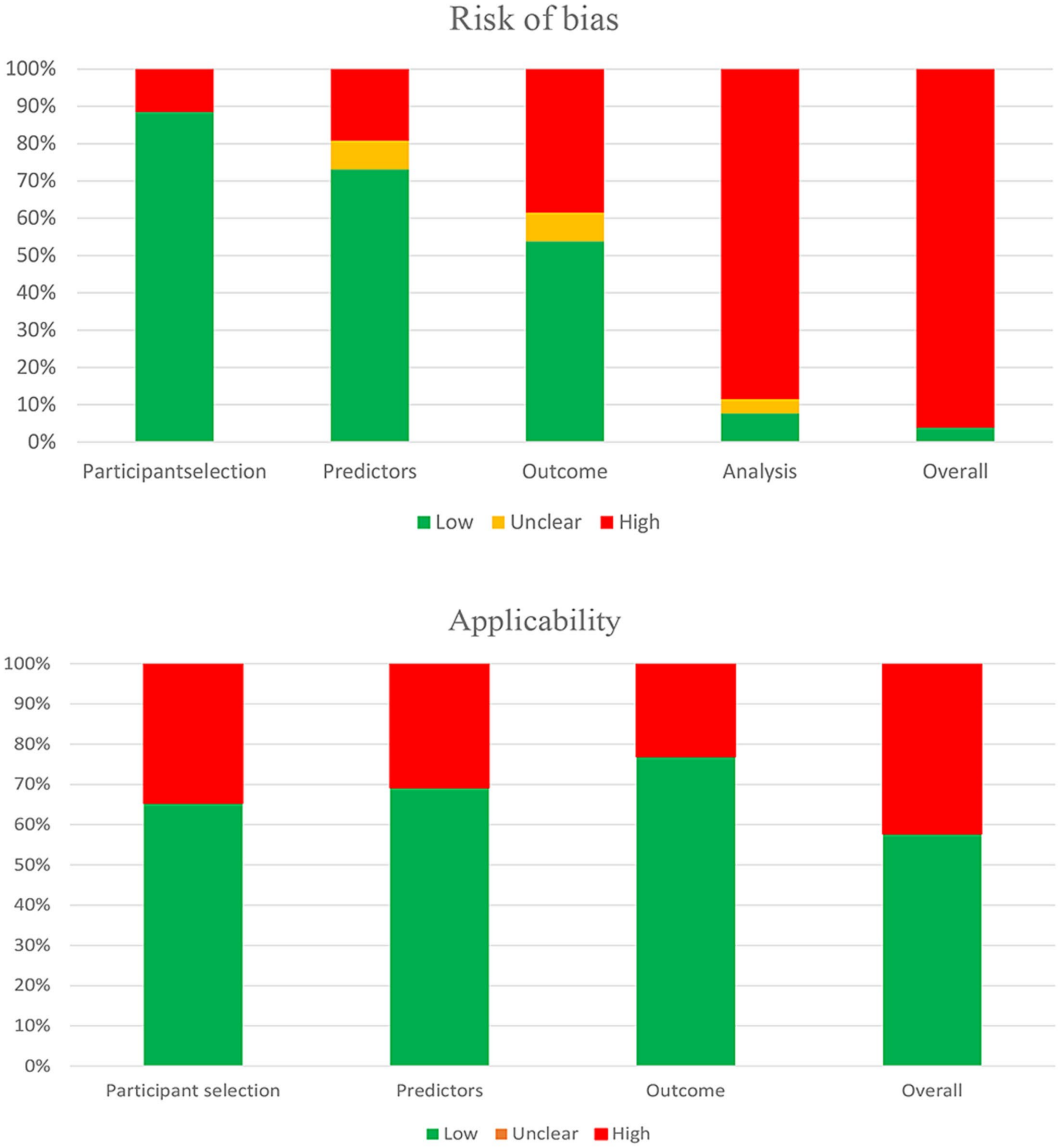
The risk of bias and applicability assessment using the PROBAST tools.

The PROBAST tool indicated high overall risk of bias in 25 studies (96.1%). For applicability, 11 studies (42.3%) exhibited high risk, while 15 studies (57.7%) were deemed low risk (Figure 3). Domain-specific bias assessments were presented as follows: (1) Participants domain: 1 study excluded participants ≥85 years and was rated as high risk of bias due to limited generalizability.; (2) Predictor domain: 5 studies were rated as high risk due to including handgrip strength as a predictor, thus introducing overestimation bias and the risk of overfitting. 1 study was rated as having unclear risk, as it lacked sufficient detail on how predictors were assessed; (3) Outcome domain: 10 studies were classified as high-risk because they failed to exclude factors such as grip strength from the definition of the results, or did not follow the international consensus on sarcopenia. 2 study was classified as having unclear risk because there was a lack of relevant information on whether the predictive factors were excluded from the result determination; (4) Analysis domain: Most models (*n* = 23) exhibited high risk due to inadequate sample sizes, arbitrary categorization of continuous variables, poor handling of missing data, reliance on univariate predictors, incomplete performance assessments, and lack of internal or external validation. Regarding applicability, 9 studies were rated high risk in the participant domain due to inconsistent target populations (e.g., younger participants or exclusion of the “oldest old” or combined with chronic diseases). In the predictors and outcomes domains, 8 studies exhibited high risk because predictors (e.g., handgrip strength) overlapped with outcome definitions.

In summary, while 96.1% of models showed high bias risk, 42.3% had acceptable applicability. These methodological flaws limited their performance and generalizability in clinical practice (Figure 3).

### Summary of AUC values in included models

**Figure 4:**
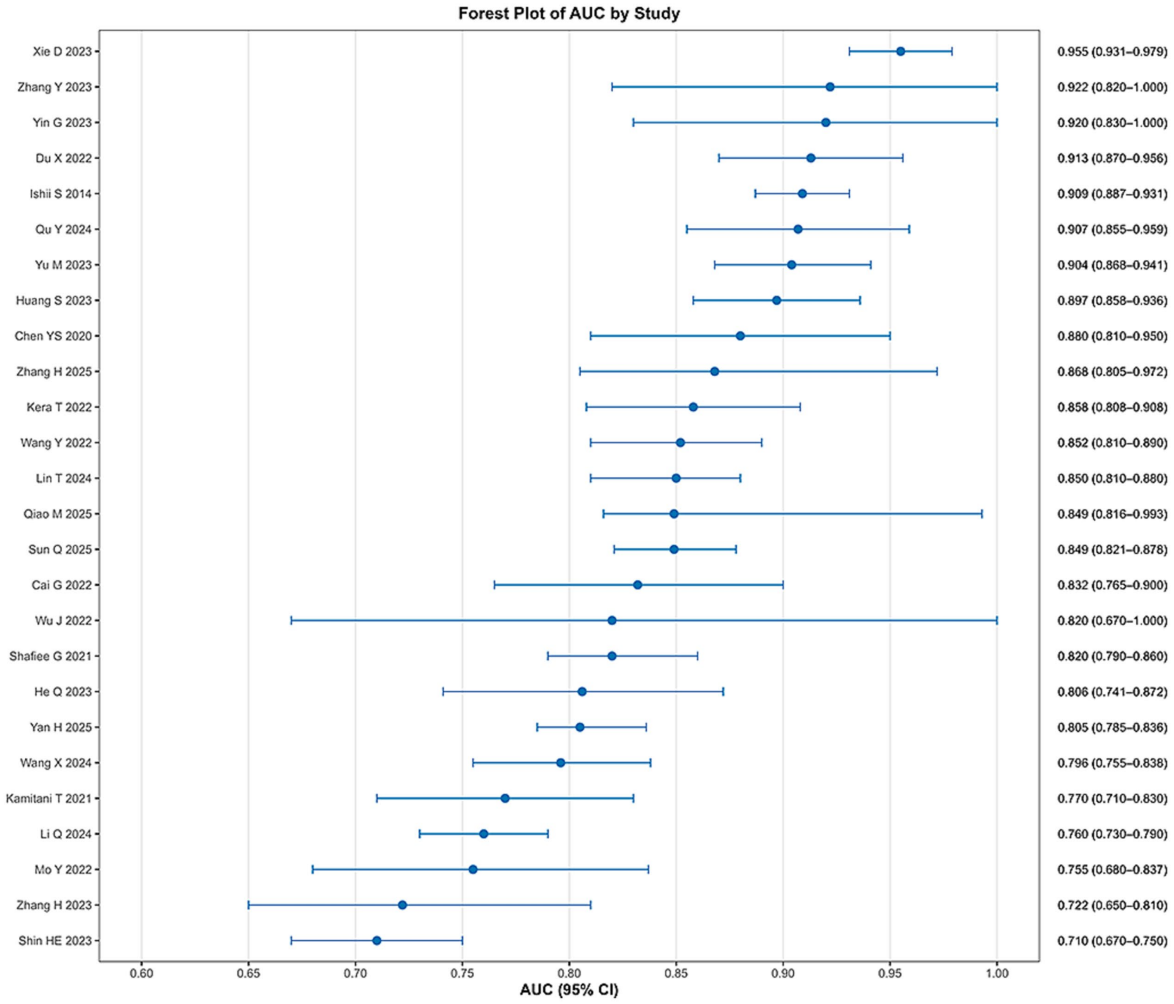
Overall summary of the AUCs of the sarcopenia prediction models included.

Due to significant heterogeneity in population, model design, validation strategies, and predictors, combined effect size or subgroup analyses were not performed. Instead, AUC values were summarized and visualized in a forest plot (Figure 4), with all models achieving AUCs above 0.70, indicating good discriminatory ability. However, the absence of external validation and comparative analyses hindered drawing conclusions on effectiveness. Methodological issues, including non-pragmatic predictor selection, missing data, inconsistent handling of continuous variables, and poor reporting, contributed to high bias risk and concern the reliability of AUC values. Although many models demonstrated promising performance, these results should be interpreted with caution. Future studies should focus on external validation and methodological improvements.

## Discussion

This systematic review conducted a comprehensive assessment of sarcopenia prediction models, highlighting significant methodological shortcomings that undermined their reliability, generalizability, and application potential in clinical settings. Most of the existing models focused on the diagnosis of current sarcopenia rather than the prediction of its future risks, and were unable to identify high-risk older people with sarcopenia development at an early stage and formulate risk-based management strategies. Moreover, these prediction models exhibited a high risk of bias due to methodological shortcomings, including the lack of external validation, inadequate handling of predictor variables and missing data, and the suboptimal application of statistical methods.

Despite the promising discrimination performance reported in most models (AUC > 0.70), the PROBAST tool revealed that 96.1% of the reviewed studies were at high risk of bias. This highlighted fundamental challenges and concerns in study design, data processing, and statistical analyses. One study exhibited a relatively low risk of bias. However, its primary focused on a highly specific patient cohort (e.g., hospitalized individuals with type 2 diabetes mellitus) might restrict its generalizability to the broader population of community-dwelling older adults. Additionally, the extent to which the model improved clinical decision-making or patient outcomes remains undetermined. The original Ishii development study (25) and the external validation by Erdogan et al. (49) indicated that this simple score—based on age, handgrip strength, and calf circumference—could achieve reasonably robust diagnostic performance for sarcopenia across diverse community-dwelling populations. However, both studies primarily evaluated the Ishii test as a cross-sectional screening tool rather than as a prognostic prediction model in selected community samples, which might limit its ability to predict future sarcopenia risk and its generalizability to other clinical settings. A critical shift from diagnostic screening to prognostic prediction is essential for the early prevention of sarcopenia. Prognostic models, such as those presented via nomograms, allow for individual risk stratification over time, enabling clinicians to implement proactive interventions long before the clinical onset of the condition (50).

This review identified several significant methodological challenges. The absence of external validation in independent populations represented a significant limitation in existing studies, as external validation was widely considered the gold standard for establishing the generalizability of predictive models. Future sarcopenia prediction research should follow the example of recent high-quality studies in other fields that utilize large, independent cohorts to confirm model performance before clinical implementation (51–54). While most current models relied on traditional logistic regression, machine learning (ML) algorithms offered a promising alternative by capturing complex, non-linear interactions between risk factors. Recent studies in other chronic diseases have demonstrated that ML-based approaches can significantly improve predictive accuracy and clinical utility, particularly when integrated into intelligent web-based assessment tools (55–57). However, the proportion of sarcopenia models at high overall risk of bias in our review (96.1%) appeared even higher than that described in other chronic disease fields (e.g., cardiovascular disease, dementia or oncology), where methodological rigor and large-scale external validation were often more established (53, 56, 57). This emphasized the need for the sarcopenia research to adopt and consistently implement the TRIPOD statement (Transparent Reporting of a multivariable prediction model for Individual Prognosis Or Diagnosis) for prediction model development, validation, and reporting (16, 58, 59).

This systematic review comprehensively evaluated sarcopenia risk prediction models for the first time, adopting a powerful methodological framework integrating PICOTS and CHARMS, which improved the validity, repeatability and overall credibility of the research results. We systematically reviewed and evaluated the current sarcopenia prediction models in various clinical settings, thereby providing additional evidence of current research deficiencies and future research directions for the primary prevention and management of sarcopenia among older adults (47, 48). Furthermore, we ensured the robustness of the research by inviting an experienced research librarian to formulate a search strategy and conducted a comprehensive search of eligible studies in four major biomedical databases. The advantages of this review also included strict screening methods, standardized data extraction, and the use of the PROBAST tool to assess the risk of bias.

However, several limitations should be acknowledged. First, only English-language publications were included in this review, potentially introducing language bias. Second, given the substantial heterogeneity among the included studies, quantitative syntheses such as meta-analysis and subgroup analysis on the reported predictive performance (AUC values) were unfeasible. Thirdly, most of the included studies lacked external validation, which hindered the ability to compare models or provide definitive recommendations for clinical adoption. Fourth, our review did not undertake a separate, systematic synthesis of external validation-only studies, which will be an important area for future research. In addition, following PROBAST guidelines, we defined an adequate sample size based on the number of participants with the outcome relative to the number of predictors. We acknowledged that in many clinical settings, especially among specific patient cohorts with low sarcopenia prevalence (ranging from 5.8 to 22.5% in our study), meeting these strict statistical requirements was a practical challenge that might necessitate multi-center collaborations. Finally, beyond clinical variables, future high-precision models could benefit from the integration of genetic risk factors and biological markers to improve the predictive accuracy and personalization of sarcopenia risk assessments (60).

## Conclusion

This systematic review identified 26 sarcopenia prediction models, mostly focusing on diagnostic study design and developed for older adults in community or clinical settings. While most existing sarcopenia prediction models were not yet recommended for general clinical application due to a high risk of bias, they might still offer valuable reference in specific contexts, such as resource-limited settings, after rigorous external validation.

## Data Availability

The original contributions presented in the study are included in the article/Supplementary material, further inquiries can be directed to the corresponding author.

## References

[1] Von Haehling S Morley JE Anker SD. An overview of sarcopenia: facts and numbers on prevalence and clinical impact. J Cachexia Sarcopenia Muscle. (2010) 1:129–33. doi: 10.1007/s13539-010-0014-2, 21475695 PMC3060646

[2] Chen LK Woo J Assantachai P Auyeung TW Chou MY Iijima K . Asian working group for sarcopenia: 2019 consensus update on sarcopenia diagnosis and treatment. J Am Med Dir Assoc. (2020) 21:e302:300–7. doi: 10.1016/j.jamda.2019.12.012 32033882

[3] Cruz-Jentoft AJ Sayer AA. Sarcopenia. Lancet. (2019) 393:2636–46. doi: 10.1016/s0140-6736(19)31138-9, 31171417

[4] Zanker J Sim M Anderson K Balogun S Brennan-Olsen SL Dent E . Consensus guidelines for sarcopenia prevention, diagnosis and management in Australia and New Zealand. J Cachexia Sarcopenia Muscle. (2023) 14:142–56. doi: 10.1002/jcsm.13115, 36349684 PMC9891980

[5] Rogeri PS Zanella R Jr Martins GL Garcia MDA Leite G Lugaresi R . Strategies to prevent sarcopenia in the aging process: role of protein intake and exercise. Nutrients. (2021) 14:10.3390/nu14010052. doi: 10.3390/nu14010052, 35010928 PMC8746908

[6] Beckwée D Delaere A Aelbrecht S Baert V Beaudart C Bruyere O . Exercise interventions for the prevention and treatment of sarcopenia. A systematic umbrella review. J Nutr Health Aging. (2019) 23:494–502. doi: 10.1007/s12603-019-1196-8, 31233069 PMC12280365

[7] Cruz-Jentoft AJ Bahat G Bauer J Boirie Y Bruyère O Cederholm T . Sarcopenia: revised European consensus on definition and diagnosis. Age Ageing. (2019) 48:601. doi: 10.1093/ageing/afz046, 31081853 PMC6593317

[8] Deer RR Akhverdiyeva L Kuo YF Volpi E. Developing a screening tool for sarcopenia in hospitalized geriatric patients: estimation of appendicular skeletal muscle mass using bioelectrical impedance. Clin Nutr. (2020) 39:2233–7. doi: 10.1016/j.clnu.2019.10.005, 31676257 PMC7153986

[9] Mo YH Su YD Dong X Zhong J Yang C Deng WY . Development and validation of a nomogram for predicting sarcopenia in community-dwelling older adults. J Am Med Dir Assoc. (2022) 23:e5:715–21. doi: 10.1016/j.jamda.2021.11.023, 34932988

[10] Tseng TG Lu CK Hsiao YH Pan S-C Tai C-J Lee M-C. Development of Taiwan risk score for sarcopenia (TRSS) for sarcopenia screening among community-dwelling older adults. Int J Environ Res Public Health. (2020) 17. doi: 10.3390/ijerph17082859, 32326323 PMC7216229

[11] Moons KGM Wolff RF Riley RD Whiting PF Westwood M Collins GS . PROBAST: a tool to assess risk of Bias and applicability of prediction model studies: explanation and elaboration. Ann Intern Med. (2019) 170:W1–w33. doi: 10.7326/m18-1377, 30596876

[12] Sayer AA Cruz-Jentoft A. Sarcopenia definition, diagnosis and treatment: consensus is growing. Age Ageing. (2022) 51. doi: 10.1093/ageing/afac220 PMC9588427 36273495

[13] Anker SD Morley JE Von Haehling S. Welcome to the ICD-10 code for sarcopenia. J Cachexia Sarcopenia Muscle. (2016) 7:512–4. doi: 10.1002/jcsm.12147, 27891296 PMC5114626

[14] Moons KG De Groot JA Bouwmeester W Moons KGM de Groot JAH Vergouwe Y . Critical appraisal and data extraction for systematic reviews of prediction modelling studies: the CHARMS checklist. PLoS Med. (2014) 11:e1001744. doi: 10.1371/journal.pmed.1001744, 25314315 PMC4196729

[15] Debray TP Damen JA Snell KI . A guide to systematic review and meta-analysis of prediction model performance. BMJ. (2017) 356:i6460. doi: 10.1136/bmj.i6460 28057641

[16] Bonnett LJ Snell KIE Collins GS Riley RD. Guide to presenting clinical prediction models for use in clinical settings. BMJ. (2019) 365. doi: 10.1136/bmj.l737 30995987

[17] Liberati A Altman DG Tetzlaff J Mulrow C Gøtzsche PC Ioannidis JP . The PRISMA statement for reporting systematic reviews and meta-analyses of studies that evaluate healthcare interventions: explanation and elaboration. BMJ. (2009) 339:339 (b2700. doi: 10.1136/bmj.b2700, 19622552 PMC2714672

[18] Chen LK Liu LK Woo J Assantachai P Auyeung T-W Bahyah KS . Sarcopenia in Asia: consensus report of the Asian working Group for Sarcopenia. J Am Med Dir Assoc. (2014) 15:95–101. doi: 10.1016/j.jamda.2013.11.025 24461239

[19] Cruz-Jentoft AJ Baeyens JP Bauer JM Boirie Y Cederholm T Landi F . Sarcopenia: European consensus on definition and diagnosis: report of the European working group on sarcopenia in older people. Age Ageing. (2010) 39:412–23. doi: 10.1093/ageing/afq034, 20392703 PMC2886201

[20] Damen JA Hooft L Schuit E Debray TP Collins GS Tzoulaki I . Prediction models for cardiovascular disease risk in the general population: systematic review. BMJ. (2016) 353:i2416. doi: 10.1136/bmj.i2416 27184143 PMC4868251

[21] González-Xuriguera CG Vergara-Merino L Garegnani L Ortiz-Muñoz L Meza N. Introduction to network meta-analysis for evidence synthesis. Medwave. (2021) 21:e8315. doi: 10.5867/medwave.2021.06.8315, 34292922

[22] Cai G Ying J Pan M Lang X Yu W Zhang Q. Development of a risk prediction nomogram for sarcopenia in hemodialysis patients. BMC Nephrol. (2022) 23:319. doi: 10.1186/s12882-022-02942-0, 36138351 PMC9502581

[23] Chen YS Cai YX Kang XR Zhou Z-h Qi X Ying C-t . Predicting the risk of sarcopenia in elderly patients with patellar fracture: development and assessment of a new predictive nomogram. PeerJ. (2020) 8:e8793. doi: 10.7717/peerj.8793, 32328345 PMC7166043

[24] Huang SW Long H Mao ZM Xiao X Chen A Liao X . A nomogram for optimizing sarcopenia screening in community-dwelling older adults: AB3C model. J Am Med Dir Assoc. (2023) 24:497–503. doi: 10.1016/j.jamda.2023.02.001, 36924796

[25] Ishii S Tanaka T Shibasaki K Ouchi Y Kikutani T Higashiguchi T . Development of a simple screening test for sarcopenia in older adults. Geriatr Gerontol Int. (2014) 14:93–101. doi: 10.1111/ggi.12197 24450566

[26] Kamitani T Wakita T Wada O Mizuno K Kurita N. U-TEST, a simple decision support tool for the diagnosis of sarcopenia in orthopaedic patients: the screening for people suffering sarcopenia in orthopedic cohort of Kobe study (SPSS-OK). Br J Nutr. (2021) 126:1323–30. doi: 10.1017/s0007114521000106, 33441195

[27] Kera T Kawai H Takahashi J Hirano H Watanabe Y Fujiwara Y . Development of a screening formula for sarcopenia using ground reaction force during sit-to-stand motion. Gait Posture. (2022) 93:177–82. doi: 10.1016/j.gaitpost.2022.02.001, 35180686

[28] Shafiee G Ostovar A Maleki Birjandi S Nabipour I Larijani B Heshmat R. Development of a simple and practical screening tool for detection of sarcopenia in older people: the Bushehr elderly health program. Front Med (Lausanne). (2021) 8. doi: 10.3389/fmed.2021.655759, 33928107 PMC8076573

[29] Shin HE Won CW Kim M. Development of multiple biomarker panels for prediction of sarcopenia in community-dwelling older adults. Arch Gerontol Geriatr. (2023) 115:115 (105115. doi: 10.1016/j.archger.2023.105115, 37422966

[30] Wang Y Zhang Y Lane NE Wu J Yang T Li J . Population-based metagenomics analysis reveals altered gut microbiome in sarcopenia: data from the Xiangya sarcopenia study. J Cachexia Sarcopenia Muscle. (2022) 13:2340–51. doi: 10.1002/jcsm.13037, 35851765 PMC9530518

[31] Zhang H Yin M Liu Q Ding F Hou L Deng Y . Machine and deep learning-based clinical characteristics and laboratory markers for the prediction of sarcopenia. Chin Med J. (2023) 136:967–73. doi: 10.1097/cm9.0000000000002633 37098831 PMC10278711

[32] Du X Chen G Zhang H Liu Y Gu F Wang Y . Development of a practical screening tool to predict sarcopenia in patients on maintenance hemodialysis. Med Sci Monit. (2022) 28:e937504. doi: 10.12659/msm.937504 36217291 PMC9569148

[33] He Q Wang X Yang C Zhuang X Yue Y Jing H . A new, alternative risk score for sarcopenia in Chinese patients with type 2 diabetes mellitus. Eur J Med Res. (2023) 28:165. doi: 10.1186/s40001-023-01127-1, 37161594 PMC10170735

[34] Wu J Lin S Guan J Wu X Ding M Shen S. Prediction of the sarcopenia in peritoneal dialysis using simple clinical information: a machine learning-based model. Semin Dial. (2023) 36:390–8. doi: 10.1111/sdi.13131 36890621

[35] Xie D Zhu Q Lu J Hu C Niu J Yu C . Development and validation of a diagnostic nomogram for sarcopenia in Chinese hemodialysis patients. Nephrol Dial Transplant. (2023) 38:1017–26. doi: 10.1093/ndt/gfac260, 36084001

[36] Yin G Qin J Wang Z Lv F Ye X. A nomogram to predict the risk of sarcopenia in older people. Medicine (Baltimore). (2023) 102:e33581. doi: 10.1097/md.0000000000033581, 37083805 PMC10118347

[37] Yu M Pan M Liang Y Li X Li J Luo L. A nomogram for screening sarcopenia in Chinese type 2 diabetes mellitus patients. Exp Gerontol. (2023) 172:112069. doi: 10.1016/j.exger.2022.112069, 36535452

[38] Zhang Y Zhu Y. Development and validation of risk prediction model for sarcopenia in patients with colorectal cancer. Front Oncol. (2023) 13:1172096. doi: 10.3389/fonc.2023.1172096, 37576879 PMC10416104

[39] Li Q Cheng H Cen W Yang T Tao S. Development and validation of a predictive model for the risk of sarcopenia in the older adults in China. Eur J Med Res[J]. (2024) 29:278. doi: 10.1186/s40001-024-01873-w 38725036 PMC11084063

[40] Lin T Liang R Song Q Liao H Dai M Jiang T . Development and validation of PRE-SARC (PREdiction of SARCopenia risk in community older adults) sarcopenia prediction model. J Am Med Dir Assoc[J]. (2024) 25:105128. doi: 10.1016/j.jamda.2024.105128 38977200

[41] Qiao M Wang H Qin M Xing T Li Y. Development and validation of a predictive model for the risk of possible sarcopenia in middle-aged and older adult diabetes mellitus in China. Front Public Health[J]. (2025) 13:1521736. doi: 10.3389/fpubh.2025.1521736 40247871 PMC12003298

[42] Qu Y Zhang L Liu Y Fu Y Wang M Liu C . Development and validation of a predictive model assessing the risk of sarcopenia in rheumatoid arthritis patients. Front Immunol[J]. (2024) 15:1437980. doi: 10.3389/fimmu.2024.1437980 39136015 PMC11317408

[43] Sun Q Shen L Liu H Lou Z Kong Q. Development and validation of a predictive model for sarcopenia risk in older Chinese adults based on key factors. BMC Geriatr[J]. (2025) 25:464. doi: 10.1186/s12877-025-06104-3 40604452 PMC12217298

[44] Wang X Gao S. Development and validation of a risk prediction model for sarcopenia in Chinese older patients with type 2 diabetes mellitus. Diabetes Metab Syndr Obes[J]. (2024) 17:4611–26. doi: 10.2147/dmso.S493903 39635500 PMC11616483

[45] Yan H Li J Li Y Xian L Tang H Zhao X . Personalised screening tool for early detection of sarcopenia in stroke patients: a machine learning-based comparative study. Aging Clin Exp Res[J]. (2025) 37:40. doi: 10.1007/s40520-025-02945-5 39979762 PMC11842499

[46] Zhang H Jin Y Che S Song Z. Nomogram models for predicting sarcopenia in elderly Asian patients with type 2 diabetes. Clinics (Sao Paulo)[J]. (2025) 80:100771. doi: 10.1016/j.clinsp.2025.100771 40915183 PMC12450744

[47] Lin T Huang X Guo D Zhao Y Song Q Liang R . Pain as a risk factor for incident sarcopenia in community-dwelling older adults: a 1-year prospective cohort study. J Am Geriatr Soc. (2023) 71:546–52. doi: 10.1111/jgs.18118, 36330882

[48] Wang X Luo Y He S Lu Y Gong Y Gao L . Age-, sex- and proximal-distal-resolved multi-omics identifies regulators of intestinal aging in non-human primates. Nat Aging. (2024) 4:414–33. doi: 10.1038/s43587-024-00572-9, 38321225 PMC10950786

[49] Erdogan T Catikkas NM Oren MM Kılıc C Karan MA Bahat G. Ishii test for screening sarcopenia: performance in community-dwelling older adults. Aging Clin Exp Res. (2022) 34:785–91. doi: 10.1007/s40520-021-01998-6, 34665450

[50] Yi X He Y Qian G Deng C Qin J Zhou X . Prognostic nomogram and epidemiological analysis for lung atypical carcinoid: a SEER database and external validation study. Cancer Med. (2024) 13:e6794. doi: 10.1002/cam4.6794, 38115788 PMC10807636

[51] Altman DG Vergouwe Y Royston P Moons KGM. Prognosis and prognostic research: validating a prognostic model. BMJ. (2009) 338:b605. doi: 10.1136/bmj.b605, 19477892

[52] Moons KG Kengne AP Grobbee DE Royston P Vergouwe Y Altman DG . Risk prediction models: II. External validation, model updating, and impact assessment. Heart. (2012) 98:691–8. doi: 10.1136/heartjnl-2011-301247, 22397946

[53] Yi X Zhang Y Cai J Hu Y Wen K Xie P . Development and external validation of machine learning-based models for predicting lung metastasis in kidney Cancer: a large population-based study. Int J Clin Pract. (2023) 2023. doi: 10.1155/2023/8001899, 37383704 PMC10299882

[54] Yi X Xu W Tang G Zhang L Wang K Luo H . Individual risk and prognostic value prediction by machine learning for distant metastasis in pulmonary sarcomatoid carcinoma: a large cohort study based on the SEER database and the Chinese population. Front Oncol. (2023) 13:1105224. doi: 10.3389/fonc.2023.1105224, 37434968 PMC10332636

[55] Xia K Chen D Jin S Yi X Luo L. Prediction of lung papillary adenocarcinoma-specific survival using ensemble machine learning models. Sci Rep. (2023) 13:14827. doi: 10.1038/s41598-023-40779-1, 37684259 PMC10491759

[56] Zhang Y Yi X Tang Z Xie P Yin N Deng Q . Using machine learning to predict lymph node metastasis in patients with renal cell carcinoma: a population-based study. Front Public Health. (2023) 11:1104931. doi: 10.3389/fpubh.2023.1104931, 37033061 PMC10080072

[57] Rong K Yi Ke Ran GLJ Zhou C Yi X. Intelligent predictive risk assessment and management of sarcopenia in chronic disease patients using machine learning and a web-based tool. Eur J Med Res. (2025) 30:345. doi: 10.1186/s40001-025-02606-3, 40301941 PMC12039279

[58] Collins GS Reitsma JB Altman DG Moons KGM. Transparent reporting of a multivariable prediction model for individual prognosis or diagnosis (TRIPOD): the TRIPOD statement. BMJ. (2015) 350:g7594. doi: 10.1136/bmj.g7594 25569120

[59] Steyerberg EW Vergouwe Y. Towards better clinical prediction models: seven steps for development and an ABCD for validation. Eur Heart J. (2014) 35:1925–31. doi: 10.1093/eurheartj/ehu207, 24898551 PMC4155437

[60] Yi X Liu E Wang Y. Post-genome-wide association study dissects genetic vulnerability and risk gene expression of Sjögren's disease for cardiovascular disease. J Transl Med. (2025) 23:531. doi: 10.1186/s12967-025-06568-2, 40350475 PMC12067732

