# Supplementary material for: The risk prediction models for sarcopenia in older adults: a systematic review and critical appraisal
Source: Front Public Health. 2026 Jan 29;14:1751954. doi: 10.3389/fpubh.2026.1751954 (PMC12896221; doi:10.3389/fpubh.2026.1751954)
Supplement: Supplementary file 1 [file Supplementary_file_1.docx]

**The risk prediction models for sarcopenia in older adults: a systematic review and critical appraisal**

**SUPPLEMENTARY MATERIAL LEGENDS**

[Supplementary Table S1 Detailed Search Strategy via OVID SP 2](#_Toc202906172)

[Supplementary Table S2 Framing the systematic review question using PICOTS guidance 4](#_Toc202906173)

[Supplementary Table S3 The reasons for exclusion after the full-text review 5](#_Toc202906174)

[Supplementary Table S4 The specific diagnostic criteria and thresholds for sarcopenia definition 7](#_Toc202906175)

[Supplementary Table S5 PRISMA Reporting Checklist 9](#_Toc202906176)

Supplementary Table S1 Detailed Search Strategy via OVID SP

| **Ovid MEDLINE(R) and Epub Ahead of Print, In-Process, In-Data-Review & Other Non-Indexed Citations, Daily and Versions <1946 to** **September 29, 2025>**  1 exp Muscular Atrophy/  2 (sarcopen* or myopen* or dynapon* or amyotroph* or myoatroph* or myophagis* or myodegenerat*).ti,ab.  3 ((muscle or muscular) adj5 (atroph* or wast* or weak* or loss* or mass or degenerat* or area?)).ti,ab.  4 1 or 2 or 3  5 exp "Sensitivity and Specificity"/  6 exp Regression Analysis/  7 area under curve/  8 exp Multivariate Analysis/  9 Regression.ti,ab.  10 (logistic adj2 model*).ti,ab.  11 (area? adj2 under adj5 curve?).ti,ab.  12 (ROC or AUC or AUCs or ACC or HSROC or SROC or AUROC).ti,ab.  13 (multivariate adj3 analysis).ti,ab.  14 5 or 6 or 7 or 8 or 9 or 10 or 11 or 12 or 13  15 4 and 14  16 ((model* or predict* or screen* or diagnostic* or tool* or risk* or SCORE*) adj6 (sarcopen* or myopen* or dynapon* or amyotroph* or myoatroph* or myophagis* or myodegenerat*)).ti,ab.  17 15 or 16  18 (community or population-based or dwelling or cohort).mp.  19 17 and 18 |
| --- |
| **Embase <1974 to September 29, 2025>**  1 exp Muscular Atrophy/  2 (sarcopen* or myopen* or dynapon* or amyotroph* or myoatroph* or myophagis* or myodegenerat*).ti,ab.  3 ((muscle or muscular) adj5 (atroph* or wast* or weak* or loss* or mass or degenerat* or area?)).ti,ab.  4 1 or 2 or 3  5 exp regression model/  6 exp area under the curve/  7 exp multivariate analysis/  8 receiver operating characteristic/  9 diagnostic test accuracy study/  10 Regression.ti,ab.  11 (logistic adj2 model*).ti,ab.  12 (area? adj2 under adj5 curve?).ti,ab.  13 (ROC or AUC or AUCs or ACC or HSROC or SROC or AUROC).ti,ab.  14 (multivariate adj3 analysis).ti,ab.  15 5 or 6 or 7 or 8 or 9 or 10 or 11 or 12 or 13 or 14  16 4 and 15  17 ((model* or predict* or screen* or diagnostic* or tool* or risk* or SCORE*) adj6 (sarcopen* or myopen* or dynapon* or amyotroph* or myoatroph* or myophagis* or myodegenerat*)).ti,ab.  18 16 or 17  19 exp animal/  20 human/  21 19 not 20  22 18 not 21  23 (community or population-based or dwelling or cohort).mp.  24 22 and 23 |
| **EBM Reviews - Cochrane Central Register of Controlled Trials < September 29, 2025>**  1 exp Muscular Atrophy/  2 (sarcopen* or myopen* or dynapon* or amyotroph* or myoatroph* or myophagis* or myodegenerat*).ti,ab.  3 ((muscle or muscular) adj5 (atroph* or wast* or weak* or loss* or mass or degenerat* or area?)).ti,ab.  4 1 or 2 or 3  5 exp "Sensitivity and Specificity"/  6 exp Regression Analysis/  7 area under curve/  8 exp Multivariate Analysis/  9 Regression.ti,ab.  10 (logistic adj2 model*).ti,ab.  11 (area? adj2 under adj5 curve?).ti,ab.  12 (ROC or AUC or AUCs or ACC or HSROC or SROC or AUROC).ti,ab.  13 (multivariate adj3 analysis).ti,ab.  14 5 or 6 or 7 or 8 or 9 or 10 or 11 or 12 or 13  15 4 and 14  16 ((model* or predict* or screen* or diagnostic* or tool* or risk* or SCORE*) adj6 (sarcopen* or myopen* or dynapon* or amyotroph* or myoatroph* or myophagis* or myodegenerat*)).ti,ab.  17 15 or 16 |
| **Science Citation Index Expanded (SCI-EXPANDED)—1900 to 2025 September 29** |
| 1 ((model* or predict* or screen* or SCORE*) near/6 (sarcopen* or myopen* or dynapon* or amyotroph* or myoatroph* or myophagis* or myodegenerat*)) or ((sarcopen* or myopen* or dynapon* or amyotroph* or myoatroph* or myophagis* or myodegenerat* or ((muscle or muscular) near/5 (atroph* or wast* or weak* or loss* or mass or degenerat* or area$))) and (regression or ROC or AUC or AUCs or ACC or HSROC or SROC or AUROC or (logistic near/2 model*) or (area$ Near/2 under near/5 curve$) or (multivariate near/3 analysis) or (sensitivity and specificity))) And community or population-based or dwelling or Cohort |

Supplementary Table S2 Framing the systematic review question using PICOTS guidance

|  | **Systematic review characteristics** |
| --- | --- |
| **1. Population** | Any older study populations enrolled to construct the sarcopenia prediction model |
| **2. Intervention** | Any diagnostic model or prognostic model to predict the possible existence or occurrence of sarcopenia, or any prediction models designed to cross-sectionally distinguish participants with sarcopenia or those at high-risk of sarcopenia incidence in the future |
| **3. Comparator** | Not applicable |
| **4. Outcome** | Presence of sarcopenia or the likelihood of sarcopenia occurrence |
| **5. Timing** | Predictors measured at any timepoint in clinical course of outcome; outcome measured without applying any specific limitation in prediction horizon |
| **6. Setting** | No limitations applied to study populations, including the general population, outpatients or inpatient diagnosed with certain chronic diseases |

CHARMS=Checklist for Critical Appraisal and Data Extraction for Systematic Reviews of Prediction Modelling Studies.

Supplementary Table S3 The reasons for exclusion after the full-text review

| **Study** | **Reason for the exclusion** | **Retrieval code** |
| --- | --- | --- |
| Lin YH 2023 | Sarcopenia scale screening studies | PMID: 36543041 |
| Zhu X 2023 | Sarcopenia scale screening studies | PMID: 36624367 |
| Ishimoto T 2022 | Sarcopenia scale screening studies | PMID: 36513470 |
| Zhou J 2022 | Sarcopenia scale screening studies | PMID: 35067707 |
| Erdogan T 2021 | Sarcopenia scale screening studies | PMID: 34665450 |
| Chen X 2021 | Sarcopenia scale screening studies | PMID: 33964883 |
| Mo YH 2021 | Sarcopenia scale screening studies | PMID: 32669238 |
| Li R 2020 | Sarcopenia scale screening studies | PMID: 33244567 |
| Mazocco L 2020 | Sarcopenia scale screening studies | PMID: 32919183 |
| Pinheiro PA 2020 | Sarcopenia scale screening studies | PMID: 30737176 |
| Bahat G 2018 | Sarcopenia scale screening studies | PMID: 30379299 |
| Yang M 2018 | Sarcopenia scale screening studies | PMID: 29477774 |
| Barreto de Lima A 2023 | Sarcopenia scale screening studies | PMID: 37474595 |
| Kera T 2023 | Sarcopenia scale screening studies | PMID: 37963490 |
| Yu S 2015 | No prediction equation for sarcopenia was available | PMID: 25239016 |
| Yoshida D | No prediction equation for sarcopenia was available | PMID: 24450560 |
| Lin YC | No prediction equation for sarcopenia was available | PMID: 32707874 |
| Lera L 2020 | No prediction equation for sarcopenia was available | PMID: 32281942 |
| Osuka Y 2020 | No prediction equation for sarcopenia was available | PMID: 32143446 |
| Ryu J 2023 | No prediction equation for sarcopenia was available | PMID: 36457204 |
| Vangelov B 2022 | No prediction equation for sarcopenia was available | PMID: 35835910 |
| Chen YL 2022 | No prediction equation for sarcopenia was available | PMID: 34873739 |
| Chen Z 2022 | No prediction equation for sarcopenia was available | PMID: 35083235 |
| Miller DK 2009 | No prediction equation for sarcopenia was available | PMID: 19181712 |
| Hwang D 2022 | No prediction equation for sarcopenia was available | PMID: 35806895 |
| Deer RR 2020 | No prediction equation for sarcopenia was available | PMID: 31676257 |
| Yin MT 2021 | No prediction equation for sarcopenia was available | PMID: 34956096 |
| Chong HJ 2025 | No prediction equation for sarcopenia was available | PMID: 40864499 |
| Yu P 2025 | No prediction equation for sarcopenia was available | PMID: 40469611 |
| Du J 2025 | No prediction equation for sarcopenia was available | PMID: 40144970 |
| Ozgur S 2023 | No prediction equation for sarcopenia was available | PMID: 37830737 |
| Kim J 2024 | No prediction equation for sarcopenia was available | Kim J 2024 |
| Liu W 2024 | No prediction equation for sarcopenia was available | PMID: 39533192 |
| Lian R 2025 | Prediction model of sarcopenia obesity | PMID: 40021576 |
| Wang Z 2025 | The same study population from CHARLS with Sun Q 2025 | PMID: 39444246 |
| Cai J 2024 | Unavailable to the original study | None |
| Gautam M | Unavailable to the original study | None |
| Shin HE 2022 | Diagnostic biomarker studies | PMID: 35963453 |
| Tang T 2022 | Diagnostic biomarker studies | PMID: 35297568 |
| Lu Y 2020 | Diagnostic biomarker studies | PMID: 30624690 |
| Zupo R 2023 | Diagnostic biomarker studies | PMID: 37110223 |
| Miyamoto K 2021 | Diagnostic biomarker studies | PMID: 33924750 |
| Osawa Y 2023 | Diagnostic biomarker studies | PMID: 37286061 |
| Zupo R 2023 | Diagnostic biomarker studies | PMID: 37110223 |
| Noorchenarboo M 2022 | Conference abstract | Not accessible |
| Petchlorlian A 2019 | Conference abstract | Not accessible |
| Semenova EA 2023 | Genetic studies | PMID: 36771461 |
| Lin S 2022 | Genetic studies | PMID: 36147639 |
| Liu X 2022 | Study protocol | PMID: 36452328 |
| Tseng TG 2022 | Invalid sarcopenia definition (lack of muscle mass) | PMID: 32326323 |
| Gray M 2016 | Invalid sarcopenia definition (lack of physical function and/or grip strength) | PMID: 26846414 |
| Guo J 2024 | Invalid sarcopenia definition (lack of physical function and/or grip strength) | PMID: 39360239 |
| Wan S 2025 | Invalid sarcopenia definition (lack of physical function and/or grip strength) | PMID: 40847308 |
| Tseng YJ 2022 | Invalid sarcopenia definition (defined merely by the cross-sectional CT slice at L3) | PMID: 36612977 |
| Hernández-Conde M 2022 | Invalid sarcopenia definition (defined merely by the cross-sectional CT slice at L3) | PMID: 35580823 |
| Tandon P 2016 | Invalid sarcopenia definition (defined merely by the cross-sectional CT slice at L3) | PMID: 27189915 |
| Kang YJ 2019 | Invalid sarcopenia definition (defined merely by skeletal muscle mass) | PMID: 31651901 |
| Kwak JY 2018 | Invalid sarcopenia definition (defined merely by skeletal muscle mass) | PMID: 29872072 |
| Liao P 2023 | Invalid sarcopenia definition (no detailed information for sarcopenia diagnosis) | PMID: 37814285 |
| Moroni A 2023 | Invalid sarcopenia definition (no detailed information for sarcopenia diagnosis) | PMID: 37960189 |

# Supplementary Table S4 The specific diagnostic criteria and thresholds for sarcopenia definition

| **Diagnostic criteria** | **Thresholds** | **References** |
| --- | --- | --- |
| **Low muscle mass** |  |  |
| BIA | 1. AWGS 2019: SMI <7.0 kg/m2 for males and<5.7 kg/m2 for females | Mo Y 2022, Cai G 2022, Kera T 2022, Du X 2022, Kamitani T 2021, Zhang H 2023, Zhang Y 2023, Xie D 2023, Yin G 2023, Wu J 2022, Huang S 2023, Wang Y 2022, Lin T 2024, Wang X 2024, Yan H 2025 |
|  | 1. AWGS 2014: SMI <7.0 kg/m2 for males and<6.0 kg/m2 for females | Chen YS 2020 |
|  | 1. AWGS 2014: SMI <7.18 kg/m2 for males and<5.73 kg/m2 for females (derived from previous studies) | He Q 2023 |
|  | 1. EWGSOP1: SMI <7.0 kg/m2 for males and<5.8 kg/m2 for females | Ishii S 2014 |
| DXA | 1. EWGSOP2: SMI <7.0 kg/m2 for males and<5.4 kg/m2 for females | Shafiee G 2021 |
|  | 1. AWGS 2019: SMI <7.0 kg/m2 for males and<5.4 kg/m2 for females | Shin HE 2023, Zhang H 2025 |
|  | 1. AWGS 2014: SMI <7.0 kg/m2 for males and<5.4 kg/m2 for females | Yu M 2023 |
| The ASM equation | 1. ASM/Ht^2^ < 5.69 kg/m^2^ for females and ASM/Ht^2^ < 6.88 kg/m^2^ for males | Li Q 2024, Qiao M 2025, Sun Q 2025 |
| **Low muscle strength** |  |  |
| HGS | 1. AWGS 2019: HGS < 28 kg in males and 18 kg in females | Mo Y 2022, Cai G 2022, Kera T 2022, Shin HE 2023, Du X 2022, Kamitani T 2021, Zhang H 2023, Zhang Y 2023, Xie D 2023, Yin G 2023, Wu J 2022, Huang S 2023, Wang Y 2022, Li Q 2024, Lin T 2024, Qiao M 2025, Sun Q 2025, Wang X 2024, Yan H 2025, Zhang H 2025 |
|  | 1. AWGS 2014: HGS < 26 kg in males or < 18 kg in females | Yu M 2023 |
|  | 1. AWGS 2014: HGS < 29.5 kg in males or < 21.2 kg in females (derived from previous studies) | He Q 2023 |
|  | 1. EWGOSP1: HGS < 30 kg in males or < 20 kg in females | Ishii S 2014 |
|  | 1. EWGOSP2: HGS < 26 kg in males or < 18 kg in females | Shafiee G 2021 |
| **Low physical performance** |  |  |
| 10-m GS | 1. AWGS 2019: GS < 1.0 m/s | Kamitani T 2021 |
| 6-m GS | 1. AWGS 2019: GS < 1.0 m/s | Mo Y 2022, Cai G 2022, Du X 2022, Zhang H 2023, Zhang Y 2023, Yin G 2023, Huang S 2023, Wang Y 2022, Li Q 2024, Lin T 2024, Qiao M 2025, Sun Q 2025, Wang X 2024, Yan H 2025 |
|  | 1. AWGS 2014: GS < 0.8 m/s | Chen YS 2020 |
| 5-m GS | 1. AWGS 2019: GS < 1.0 m/s | Kera T 2022 |
|  | 1. EWGOSP1: GS < 1.26 m/s | Ishii S 2014 |
| 4.57-m GS | 1. EWGOSP2: GS < 0.8 m/s | Shafiee G 2021 |
| 4-m GS | 1. AWGS 2019: GS < 1.0 m/s | Shin HE 2023, Xie D 2023 |
|  | 1. AWGS 2014: GS < 0.8 m/s | Yu M 2023 |
| SPPB | 1. AWGS 2019: SPPB $\leq$9 score | Shin HE 2023, Zhang H 2023, Wang Y 2022, Qiao M 2025 |
| 5-CST | 1. AWGS 2019: 5-CST$\geq$12s | Shin HE 2023, Wu J 2022, Wang Y 2022, Li Q 2024, Lin T 2024, Qiao M 2025, Sun Q 2025 |

Abbreviation：EWGSOP1, European Working Group on Sarcopenia in Older People 2010 consensus; EWGOSP2, the revised 2018 European Working Group on Sarcopenia in Older People; AWGS 2014, Asian Working Group for Sarcopenia (AWGS) 2014 consensus; AWGS 2019, Asian Working Group for Sarcopenia (AWGS) 2019 consensus; BIA: bioelectrical impedance device; DXA: dual-energy X-ray absorptiometry; HGS, hand grip strength; 6-m GS, 6-meter gait speed; GS, gait speed; 5-CST, 5-time chair stand test; SPPB, short physical performance battery; SMI, skeletal muscle mass index; SMI, skeletal mass index; The ASM equation: ASM = 0.193 * weight (kg) + 0.107 * height(cm)− 4.157 * sex − 0.037 * age (years) − 2.631. The detailed information about sarcopenia definition of Qu Y 2024 was unreported.

Supplementary Table S5 PRISMA Reporting Checklist

| **Section and Topic** | **Item #** | **Checklist item** | **Location where item is reported** |
| --- | --- | --- | --- |
| **TITLE** | | |  |
| Title | 1 | Identify the report as a systematic review. | Page 1 |
| **ABSTRACT** | | |  |
| Abstract | 2 | See the PRISMA 2020 for Abstracts checklist. | Page 1 |
| **INTRODUCTION** | | |  |
| Rationale | 3 | Describe the rationale for the review in the context of existing knowledge. | Page 2 |
| Objectives | 4 | Provide an explicit statement of the objective(s) or question(s) the review addresses. | Page 2 |
| **METHODS** | | |  |
| Eligibility criteria | 5 | Specify the inclusion and exclusion criteria for the review and how studies were grouped for the syntheses. | Page 2 |
| Information sources | 6 | Specify all databases, registers, websites, organisations, reference lists and other sources searched or consulted to identify studies. Specify the date when each source was last searched or consulted. | Page 2-3 |
| Search strategy | 7 | Present the full search strategies for all databases, registers and websites, including any filters and limits used. | Page 2 |
| Selection process | 8 | Specify the methods used to decide whether a study met the inclusion criteria of the review, including how many reviewers screened each record and each report retrieved, whether they worked independently, and if applicable, details of automation tools used in the process. | Page 3 |
| Data collection process | 9 | Specify the methods used to collect data from reports, including how many reviewers collected data from each report, whether they worked independently, any processes for obtaining or confirming data from study investigators, and if applicable, details of automation tools used in the process. | Page 3 |
| Data items | 10a | List and define all outcomes for which data were sought. Specify whether all results that were compatible with each outcome domain in each study were sought (e.g. for all measures, time points, analyses), and if not, the methods used to decide which results to collect. | Page 3 |
|  | 10b | List and define all other variables for which data were sought (e.g. participant and intervention characteristics, funding sources). Describe any assumptions made about any missing or unclear information. | Page 3 |
| Study risk of bias assessment | 11 | Specify the methods used to assess risk of bias in the included studies, including details of the tool(s) used, how many reviewers assessed each study and whether they worked independently, and if applicable, details of automation tools used in the process. | Page 2-4 |
| Effect measures | 12 | Specify for each outcome the effect measure(s) (e.g. risk ratio, mean difference) used in the synthesis or presentation of results. | Page 3-4 |
| Synthesis methods | 13a | Describe the processes used to decide which studies were eligible for each synthesis (e.g. tabulating the study intervention characteristics and comparing against the planned groups for each synthesis (item #5)). | Page 3-4 |
|  | 13b | Describe any methods required to prepare the data for presentation or synthesis, such as handling of missing summary statistics, or data conversions. | Page 3-4 |
|  | 13c | Describe any methods used to tabulate or visually display results of individual studies and syntheses. | Page 3-4 |
|  | 13d | Describe any methods used to synthesize results and provide a rationale for the choice(s). If meta-analysis was performed, describe the model(s), method(s) to identify the presence and extent of statistical heterogeneity, and software package(s) used. | None |
|  | 13e | Describe any methods used to explore possible causes of heterogeneity among study results (e.g. subgroup analysis, meta-regression). | None |
|  | 13f | Describe any sensitivity analyses conducted to assess robustness of the synthesized results. | None |
| Reporting bias assessment | 14 | Describe any methods used to assess risk of bias due to missing results in a synthesis (arising from reporting biases). | None |
| Certainty assessment | 15 | Describe any methods used to assess certainty (or confidence) in the body of evidence for an outcome. | None |
| **RESULTS** | | |  |
| Study selection | 16a | Describe the results of the search and selection process, from the number of records identified in the search to the number of studies included in the review, ideally using a flow diagram. | Page 4 |
|  | 16b | Cite studies that might appear to meet the inclusion criteria, but which were excluded, and explain why they were excluded. | Page 4, Appendix Table S3 |
| Study characteristics | 17 | Cite each included study and present its characteristics. | Page 4 |
| Risk of bias in studies | 18 | Present assessments of risk of bias for each included study. | Page 10, Figure 3 |
| Results of individual studies | 19 | For all outcomes, present, for each study: (a) summary statistics for each group (where appropriate) and (b) an effect estimate and its precision (e.g. confidence/credible interval), ideally using structured tables or plots. | Page 4-10 |
| Results of syntheses | 20a | For each synthesis, briefly summarise the characteristics and risk of bias among contributing studies. | Page 4-10 |
|  | 20b | Present results of all statistical syntheses conducted. If meta-analysis was done, present for each the summary estimate and its precision (e.g. confidence/credible interval) and measures of statistical heterogeneity. If comparing groups, describe the direction of the effect. | Page 4-10 |
|  | 20c | Present results of all investigations of possible causes of heterogeneity among study results. | Page 4-10 |
|  | 20d | Present results of all sensitivity analyses conducted to assess the robustness of the synthesized results. | None |
| Reporting biases | 21 | Present assessments of risk of bias due to missing results (arising from reporting biases) for each synthesis assessed. | None |
| Certainty of evidence | 22 | Present assessments of certainty (or confidence) in the body of evidence for each outcome assessed. | None |
| **DISCUSSION** | | |  |
| Discussion | 23a | Provide a general interpretation of the results in the context of other evidence. | Page 10 |
|  | 23b | Discuss any limitations of the evidence included in the review. | Page 13 |
|  | 23c | Discuss any limitations of the review processes used. | Page 13 |
|  | 23d | Discuss implications of the results for practice, policy, and future research. | Page 10-13 |
| **OTHER INFORMATION** | | |  |
| Registration and protocol | 24a | Provide registration information for the review, including register name and registration number, or state that the review was not registered. | None |
|  | 24b | Indicate where the review protocol can be accessed, or state that a protocol was not prepared. | None |
|  | 24c | Describe and explain any amendments to information provided at registration or in the protocol. | None |
| Support | 25 | Describe sources of financial or non-financial support for the review, and the role of the funders or sponsors in the review. | Page 13 |
| Competing interests | 26 | Declare any competing interests of review authors. | None |
| Availability of data, code and other materials | 27 | Report which of the following are publicly available and where they can be found: template data collection forms; data extracted from included studies; data used for all analyses; analytic code; any other materials used in the review. | None |

*From:*  Page MJ, McKenzie JE, Bossuyt PM, Boutron I, Hoffmann TC, Mulrow CD, et al. The PRISMA 2020 statement: an updated guideline for reporting systematic reviews. BMJ 2021;372:n71. doi: 10.1136/bmj.n71. For more information, visit: <http://www.prisma-statement.org/>
